# Assessing the Economic Impact of Cancer Care: A Study on Out‐of‐Pocket Expenditures and Utilization in South Korea

**DOI:** 10.1002/cam4.70593

**Published:** 2025-02-18

**Authors:** Do Hee Kim, Yejin Kim, Jun Su Park, Sang Gyu Lee, Hyuk‐Jae Chang, Tae Hyun Kim

**Affiliations:** ^1^ Department of Public Health, Graduate School Yonsei University Seoul Korea; ^2^ Department of Preventive Medicine Yonsei University College of Medicine Seoul Korea; ^3^ Department of Cardiology Yonsei University College of Medicine Seoul Korea; ^4^ Department of Healthcare Management Graduate School of Public Health, Yonsei University Seoul Korea

**Keywords:** cancer, economic impact, out‐of‐pocket expenditure, utilization

## Abstract

**Introduction:**

Cancer remains one of the leading causes of mortality and financial distress worldwide. In South Korea, the government introduced a benefit extension system in 2013 aimed at mitigating the financial strain associated with cancer treatment. However, cancer patients continue to bear significant out‐of‐pocket (OOP) expenses. This study aims to quantify the incremental healthcare utilization and OOP expenditures incurred by cancer patients in South Korea.

**Methods:**

Utilizing data from the 2019 Korean Health Panel (KHP), we assessed cancer‐related healthcare utilization and OOP expenditures. A generalized linear regression model, adjusted for demographic and socioeconomic variables, was employed. Healthcare utilization was measured by hospital admissions, outpatient visits, and emergency room (ER) visits, while OOP expenditures encompassed services including both covered and not covered by the National Health Insurance (NHI) system.

**Results:**

Cancer patients experienced 0.39 more hospitalizations, 4.91 additional outpatient visits, and 0.11 more ER visits annually compared to non‐cancer patients. Their incremental OOP expenses amounted to $482.8 per year, with $340.2 attributable to inpatient services. Notable variations in healthcare utilization and expenditures were observed across different cancer types.

**Discussion:**

Despite the implementation of the benefit extension system, cancer patients continue to face considerable OOP expenses, particularly for inpatient care. With cancer incidence expected to rise, there is a pressing need for more comprehensive healthcare policies that alleviate the financial burden and prioritize cost‐effective treatments for cancer patients.

**Conclusion:**

This study underscores the substantial economic impact of cancer on South Korean patients. Expanding the benefit extension system and promoting cost‐effective care strategies are critical to easing the growing financial pressures on cancer patients.

## 1 Introduction

Cancer is one of the leading causes of death globally, responsible for nearly 10 million deaths in 2020, accounting for roughly one in every six deaths [1]. The incidence of cancer continues to rise, placing increasing pressure on healthcare systems worldwide [2]. Beyond the physical and emotional toll, a cancer diagnosis is often accompanied by significant financial hardship [3, 4, 5]. Historically, cancer has been among the most expensive medical conditions to treat [6], with healthcare spending on cancer care rising in recent years [7]. As a result, many cancer survivors report difficulties in paying medical bills, experiencing financial distress, and in some cases, delaying or foregoing care [8].

To accurately estimate the additional healthcare utilization and out‐of‐pocket (OOP) expenditures borne by cancer patients, an incremental approach is frequently used. This method, leveraging regression analysis, isolates the incremental burden by comparing individuals with and without cancer, providing a more precise assessment of utilization and financial impact [9, 10, 11]. Previous studies have demonstrated the high disease burden and financial strain associated with cancer, driven by advancements in treatments such as targeted therapies, immunotherapies, and sophisticated imaging techniques, as well as longer treatment durations and the use of multiple treatment combinations [12, 13, 14].

In South Korea, the National Health Insurance (NHI) system provides near‐universal coverage, insuring approximately 97% of the population, while the remaining 3% are covered under the Medical Aid Program [15]. The NHI reduces the financial burden of healthcare by covering a substantial portion of inpatient and outpatient services, diagnostic tests, and prescription medications [16]. However, patients are still responsible for co‐payments, which typically range from 20% to 50% of covered services [16]. In addition, services not covered by NHI, including certain advanced treatments and specialized drugs, require full OOP payments, contributing significantly to financial hardship [17].

In response to these challenges, South Korea implemented a benefit extension system in October 2013 to reduce the financial burden on patients diagnosed with four major disease groups—cardiovascular diseases, cerebrovascular diseases, cancer, and intractable diseases [18]. Despite this effort, the intensity of healthcare utilization and rising cancer treatment costs continue to increase, signaling a growing economic burden on both the healthcare system and cancer patients [19, 20, 21, 22].

Approximately 75% of South Korean households enroll in private health insurance (PHI) to supplement NHI coverage, as it helps offset expenses for non‐covered services and high OOP expenses [23]. Prescription medications, although partially covered by NHI, often incur co‐payments of 35%–40%, and certain high‐cost drugs remain fully uncovered [24]. These gaps highlight the significance of studying OOP expenditures, including both covered and non‐covered services, to understand the financial challenges faced by cancer patients comprehensively.

While most studies have concentrated on healthcare payments covered by the National Health Insurance (NHI) system and patient copayments, there has been limited focus on the total OOP expenses faced by patients. This includes payments for services not covered by NHI, which can contribute significantly to the financial strain experienced by cancer patients. Therefore, this study provides valuable insights by examining OOP expenditures from the patient's perspective, including both covered and non‐covered services under NHI.

The primary objective of this study was to estimate the incremental OOP expenditures associated with cancer using an incremental expenditures approach. Given the variation in cancer incidence and treatment across different cancer types, this study also aimed to categorize and analyze healthcare utilization and OOP expenditures specific to each cancer type.

## 2 Methods

### 2.1 Study Population and Data Collection

This study utilized data from the 2019 Korean Health Panel (KHP), a survey conducted by the Korea Institute for Health and Social Affairs (KIHSA) in collaboration with the National Health Insurance Service (NHIS). The KHP is a national public database (https://www.khp.re.kr) that includes an identification number for each household and member. However, the number is not associated with any personal identifying information, and the data collection system and database were designed to protect respondents' confidentiality. The KHP includes secondary data to gather and provide household and individual‐level scientific data on health service use, expenditure, and health behaviors. The KHP employed a two‐stage clustered probability sampling method, drawing participants from a population frame established by Statistics Korea. The final study population included households and their members residing in 17 cities and provinces across South Korea, representing approximately 8500 households.

The KHP collects household and individual‐level data on health service utilization, health‐related expenditures, and behaviors. Information on diseases of interest, including cancer and other chronic conditions, is based on self‐reported physician diagnoses through structured questionnaires. Data collection was performed through both interviews and diary methods, ensuring accuracy in recall. The survey included detailed questionnaires administered to both households and individual members. The KHP has received ethical approval from KIHSA, and all participants provided informed consent before participation. A total of 13,832 participants were analyzed after excluding those with missing or incomplete data.

### 2.2 Variables and Measures

#### 2.2.1 Dependent Variables

Incremental healthcare utilization and OOP expenditures were assessed over a 1‐year period. These measures allowed for a comprehensive comparison of the economic burden between two groups: those with a cancer diagnosis and those without a history of cancer. To maintain a healthcare‐focused scope, direct non‐medical costs (e.g., transportation, caregiver expenses) were excluded from the analysis.

The primary outcome measures included 1‐year incremental healthcare utilization, specifically for hospital admissions, emergency room (ER) visits, and outpatient visits. Additionally, incremental OOP expenditures were calculated based on hospital admissions, ER visits, outpatient services, and prescription medications. OOP expenditures were defined as the financial burden for services not covered by the National Health Insurance (NHI) and statutory OOP expenditures. To standardize expenditures, the Korean Won (KRW) was converted into US dollars (USD) using an exchange rate of 1332.20 KRW per 1 USD, as of July 3, 2024. This exchange rate was applied consistently throughout the analysis.

#### 2.2.2 Variable of Interest

The primary variable of interest, a history of cancer diagnosis, was selected to differentiate healthcare utilization and OOP expenditures between individuals with and without cancer. This approach enables the analysis to isolate the economic impact of cancer while controlling for demographic, socioeconomic, and health‐related factors. The KHP categorizes cancers into several types: gastric, colorectal, lung, breast, cervical, thyroid, and others. For this study, participants were classified into the cancer group if they had at least one of the above‐mentioned diagnoses. Those without a history of these cancers were categorized into the non‐cancer group. The data do not include information on the treatment status of cancer patients, such as whether they were undergoing active treatment at the time of the survey or the time elapsed since completion of treatment. The non‐cancer group may include individuals with other chronic conditions, such as diabetes or hypertension, and does not consist solely of completely healthy individuals.

### 2.3 Covariates

A range of demographic, socio‐economic, and health‐related covariates were included in the analysis to control for potential confounding factors. In our study, the covariates were sex (male, female), age groups (≤ 49 years, 50–64 years, 65–79 years, and ≥ 80 years), marital status (not married, married, others), education status (elementary school or below, middle school, high school, college or above), insurance type (medical aid, national health insurance), private health insurance (PHI) ownership (yes, no), household income levels (quartiles: Q1, Q2, Q3, Q4), occupation (white collar, pink collar, blue collar, none), smoking status (yes, no), high‐risk alcohol consumption (yes, no), chronic conditions, such as hypertension (yes, no), and diabetes (yes, no).

### 2.4 Statistical Analysis

This study employed a cross‐sectional design using data from the 2019 KHP, a nationally representative dataset. The analysis focused on comparing healthcare utilization and OOP expenditures between individuals with and without a history of cancer.

Given that the KHP dataset was obtained using systematic sampling, weighted values were applied to the statistical analyses to ensure population‐level representativeness. To compare healthcare utilization and OOP expenditures between participants with and without cancer, boxplots were created to visualize the data distribution, and the Rao–Scott chi‐square test was utilized.

A generalized linear regression model was employed to adjust for the covariates listed above. A log‐link function was chosen for the GLM to accurately capture the relationship between the covariates and the dependent variable, while accommodating the right‐skewed nature of the data. The normal distribution was selected as the family for the dependent variable, as it was determined to best align with the continuous nature of the healthcare utilization and expenditure data in this study. This approach, leveraging regression analysis to isolate the incremental burden of cancer, aligns with methods commonly used in the literature to quantify the economic impact of diseases [9, 10, 11].

Model diagnostics were conducted to validate the choice of link function and distribution family. Residual plots were examined to assess the adequacy of the model fit, including checks for systematic patterns and heteroscedasticity. Standardized Pearson residuals, deviance residuals, and Cook's distance were evaluated to identify potential outliers and influence points. The diagnostics confirmed that the log‐link and normal distribution provided an appropriate fit for the data.

A generalized estimating equation (GEE) with a robust standard error was used to mitigate the risk of overestimating standard errors in the parameter estimates. Sensitivity analyses were conducted to evaluate incremental OOP expenditures, stratified by cancer type. All statistical analyses were performed using SAS version 9.4 (SAS Institute Inc., Cary, NC, USA), and the figures were created using R. A *p* value of less than 0.05 was considered statistically significant.

## 3 Results

### 3.1 Distribution of Participants by Characteristics

The characteristics of the study participants, stratified by cancer type and non‐cancer group, are summarized in Table 1. Among 13,832 participants, 579 were cancer patients distributed across seven cancer types. Males predominated in gastric (71.4%), colorectal (61.8%), and lung cancer (73.2%) groups, while breast, cervical, and thyroid cancer patients were predominantly female (100.0%, 100.0%, and 89.7%, respectively). Thyroid cancer was more prevalent in younger participants (19.0% aged ≤ 49 years), while lung cancer was most common among those aged 65–79 years (68.3%). Private health insurance ownership varied, with the highest rates in colorectal (69.1%) and lung cancer (63.4%) groups, while lower‐income households (Q1) were more prevalent among gastric (51.4%) and colorectal cancer (44.1%) patients. Smoking and alcohol use differed across groups, with high‐risk alcohol consumption exceeding 90% in all cancer groups. These results highlight demographic and socioeconomic differences across cancer types, providing context for further analysis.

**TABLE 1:** Characteristics of study participants by cancer type and non‐cancer group.

Variable	Non‐cancer		Gastric cancer		Colorectal cancer		Lung cancer		Breast cancer		Cervical cancer		Thyroid cancer		Other cancer	
	*N*	%	*N*	%	*N*	%	*N*	%	*N*	%	*N*	%	*N*	%	*N*	%
Total	13,253	100.0	70	100	68	100	41		71		26		116		187	
Sex																
Male	6085	45.9	50	71.4	42	61.8	30	73.2	0	0.0	0	0.0	12	10.3	119	63.6
Female	7168	54.1	20	28.6	26	38.2	11	26.8	71	100.0	26	100.0	104	89.7	68	36.4
Age group																
≤ 49	5956	44.9	0	0.0	0	0.0	0	0.0	11	15.5	4	15.4	22	19.0	14	7.5
50–64	3329	25.1	21	30.0	13	19.1	8	19.5	30	42.3	9	34.6	45	38.8	50	26.7
64–79	3313	25.0	37	52.9	40	58.8	28	68.3	26	36.6	12	46.2	48	41.4	98	52.4
> 80	655	4.9	12	17.1	15	22.1	5	12.2	4	5.6	1	3.8	1	0.9	25	13.4
Marital status																
Not married	3433	25.9	2	2.9	0	0.0	0	0.0	3	4.2	1	3.8	4	3.4	12	6.4
Married	7910	59.7	54	77.1	50	73.5	32	78.0	49	69.0	18	69.2	88	75.9	136	72.7
Others	1910	14.4	14	20.0	18	26.5	9	22.0	19	26.8	7	26.9	24	20.7	39	20.9
Education status																
Elementary school or below	3992	30.1	23	32.9	28	41.2	21	51.2	18	25.4	11	42.3	35	30.2	67	35.8
Middle school	1763	13.3	18	25.7	14	20.6	9	22.0	11	15.5	6	23.1	16	13.8	43	23.0
High school	3608	27.2	24	34.3	16	23.5	9	22.0	29	40.8	5	19.2	39	33.6	49	26.2
College or above	3890	29.4	5	7.1	10	14.7	2	4.9	13	18.3	4	15.4	26	22.4	28	15.0
Insurance type																
Medical Aid	507	3.8	4	5.7	5	7.4	4	9.8	4	5.6	0	0.0	7	6.0	15	8.0
National Health Insurance	12,746	96.2	66	94.3	63	92.6	37	90.2	67	94.4	26	100.0	109	94.0	172	92.0
Private health insurance (PHI) ownership																
Yes	5209	39.3	38	54.3	47	69.1	26	63.4	19	26.8	9	34.6	38	32.8	100	53.5
No	8044	60.7	32	45.7	21	30.9	15	36.6	52	73.2	17	65.4	78	67.2	87	46.5
Household income level																
Q1	3308	25.0	36	51.4	30	44.1	14	34.1	27	38.0	10	38.5	39	33.6	79	42.2
Q2	3304	24.9	18	25.7	19	27.9	13	31.7	19	26.8	7	26.9	28	24.1	60	32.1
Q3	3319	25.0	11	15.7	9	13.2	11	26.8	11	15.5	6	23.1	28	24.1	33	17.6
Q4	3322	25.1	5	7.1	10	14.7	3	7.3	14	19.7	3	11.5	21	18.1	15	8.0
Occupation																
White collar	2047	15.4	4	5.7	0	0.0	1	2.4	5	7.0	3	11.5	16	13.8	7	3.7
Pink collar	1406	10.6	4	5.7	1	1.5	2	4.9	7	9.9	3	11.5	19	16.4	12	6.4
Blue collar	3615	27.3	29	41.4	20	29.4	7	17.1	12	16.9	4	15.4	30	25.9	55	29.4
None	6185	46.7	33	47.1	47	69.1	31	75.6	47	66.2	16	61.5	51	44.0	113	60.4
Smoking status																
Never smoked	9165	69.2	25	35.7	41	60.3	17	41.5	69	97.2	23	88.5	104	89.7	100	53.5
Former smoker	2263	17.1	37	52.9	23	33.8	20	48.8	2	2.8	0	0.0	11	9.5	69	36.9
Current smoker	1825	13.8	8	11.4	4	5.9	4	9.8	0	0.0	3	11.5	1	0.9	18	9.6
High‐risk alcohol consumption																
Yes	11,786	88.9	66	94.3	64	94.1	39	95.1	67	94.4	25	96.2	114	98.3	178	95.2
No	1269	9.6	4	5.7	3	4.4	2	4.9	0	0.0	1	3.8	1	0.9	7	3.7
Hypertension																
Yes	9891	74.6	42	60.0	31	45.6	18	43.9	44	62.0	17	65.4	66	56.9	113	60.4
No	3362	25.4	28	40.0	37	54.4	23	56.1	27	38.0	9	34.6	50	43.1	74	39.6
Diabetes																
Yes	11,754	88.7	57	81.4	51	75.0	29	70.7	63	88.7	21	80.8	88	75.9	145	77.5
No	1499	11.3	13	18.6	17	25.0	12	29.3	8	11.3	5	19.2	28	24.1	42	22.5

### 3.2 Differences in Annual Healthcare Utilization and OOP Expenditures

**FIGURE 1:**
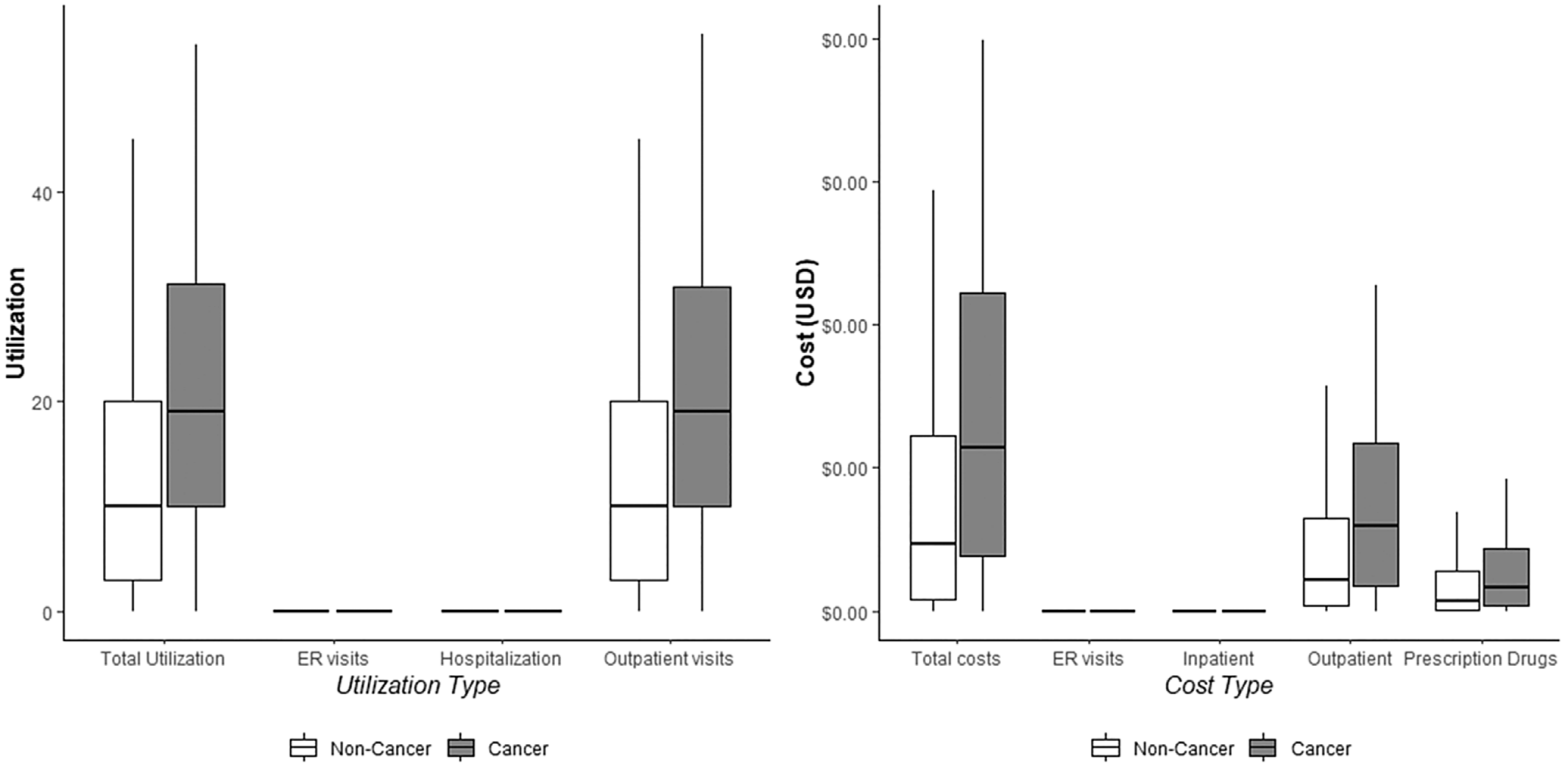
Comparison of healthcare utilization and costs between cancer and non‐cancer patients.

Table 2 and Figure 1 present the average differences in annual healthcare utilization. The cancer group experienced significantly higher utilization, with an average of 0.47 additional hospitalizations (*p* < 0.0001), 11.58 more outpatient visits (*p* < 0.0001), and 0.12 more ER visits (*p* = 0.0002) compared to the non‐cancer group. Overall, total healthcare utilization was 12.16 visits higher in the cancer group (*p* < 0.0001).

**TABLE 2:** Differences in annual healthcare utilization.

Utilization category	Cancer		Non‐cancer		Mean difference	*p*
	Mean	95% CI	Mean	95% CI		
Hospitalization	0.59	0.45–0.72	0.12	0.11–0.13	0.47	< 0.0001
Outpatient visits	25.75	22.95–28.56	14.18	13.79–14.56	11.58	< 0.0001
ER visits	0.20	0.13–0.27	0.08	0.08–0.09	0.12	0.0002
Total utilization	26.54	23.69–29.39	14.38	14–14.77	12.16	< 0.0001

Table 3 and Figure 1 summarize the differences in annual expenditures. The cancer group incurred significantly higher inpatient expenditures ($398.1; *p* < 0.0001), outpatient expenditures ($206.2; *p* < 0.0001), ER visit expenditures ($2.2; *p* < 0.0001), and total expenditures ($680.3; *p* < 0.0001) relative to the non‐cancer group.

**TABLE 3:** Differences in annual OOP expenditures.

OOP costs category	Cancer		Non‐cancer		Mean difference	*p*
	Mean ($)	95% CI	Mean ($)	95% CI		
Inpatient	495.8	348.6–642.9	97.7	85.9–109.6	398.1	< 0.0001
Outpatient	490.5	421.6–559.3	284.3	271.2–297.4	206.2	< 0.0001
ER visits	7.1	4.0–10.3	4.9	4.2–5.6	2.2	< 0.0001
Prescription drugs	144.4	121.8–167.1	70.3	67.9–72.7	74.1	< 0.0001
Total costs	1137.5	956.7–1318.4	457.2	437.4–477.0	680.3	< 0.0001

### 3.3 Association Between Cancer and Incremental Annual Healthcare Utilization

Table 4 illustrates the relationship between cancer and annual incremental healthcare utilization, adjusted for covariates such as age, sex, socioeconomic status, and comorbidities (see footnote a for details). Cancer was significantly associated with an increase in annual hospitalizations (0.39 admissions; *p* < 0.0001), as well as higher annual outpatient visits (4.91 visits; *p* < 0.0001) and ER visits (0.11 visits; *p* < 0.0001). Overall, total healthcare utilization was notably higher for individuals with cancer, with an increase of 5.40 visits (*p* < 0.0001).

**TABLE 4:** Association between cancer and annual incremental utilization and OOP expenditures.

Utilization category	*β* (Incremental utilization)	95% CI	*p*	Costs category	*β* (Incremental OOP)	95% CI	*p*
Hospitalization	0.39	0.33–0.45	< 0.0001	Inpatient	340.2	277.1–403.3	< 0.0001
Outpatient visits	4.91	3.06–6.76	< 0.0001	Outpatient	121.8	32.2–180.4	< 0.0001
ER visits	0.11	0.07–0.14	< 0.0001	ER visits	1.1	−2.4–4.5	0.5554
				Prescription drugs	19.7	9.0–30.4	0.0003
Total utilization	5.40	3.54–7.27	< 0.0001	Total costs	482.8	388.6–577.0	< 0.0001

^a^ Incremental values represent the difference in predicted means between the cancer and non‐cancer groups, adjusted for covariates.

Model diagnostics confirmed the appropriateness of the normal distribution and identity link function for healthcare utilization models. Scaled deviance and Pearson's chi‐square values were close to 1.0 for hospitalizations (1.0018), outpatient visits (1.0018), ER visits (1.0018), and total visits (1.0018), indicating good model fit. Residual plots revealed no significant patterns, and Cook's distance values, such as for outpatient visits (mean = 0.0028), were well below the threshold of 0.5, suggesting no undue influence from specific observations.

### 3.4 Association Between Cancer and Incremental Annual OOP Expenditures

Table 4 also highlights the association between cancer and incremental annual OOP expenditures, adjusted for the same covariates. Individuals with cancer incurred significantly higher total annual expenditures ($482.8; *p* < 0.0001) compared to those without cancer. Cancer patients also faced higher annual expenses per visit, including inpatient expenditures ($340.2; *p* < 0.0001) and outpatient expenditures ($121.8; *p* < 0.0001). While annual ER expenditures were slightly higher for cancer patients ($1.1; *p* = 0.5554), this difference was not statistically significant. Additionally, annual prescription drug expenditures were significantly elevated for individuals with cancer ($19.7; *p* < 0.0001).

Model diagnostics supported the appropriateness of the normal distribution and identity link function for cost models. Scaled deviance and Pearson's chi‐square values for inpatient (1.0018), outpatient (1.0018), ER (1.0018), and total expenditures (1.0018) indicated good fit. Residual analysis showed no systematic patterns, and Cook's distance values, such as for total costs (mean = 0.0035), demonstrated no evidence of influential observations.

### 3.5 Results of Subgroup Analysis by Cancer Type

**FIGURE 2:**
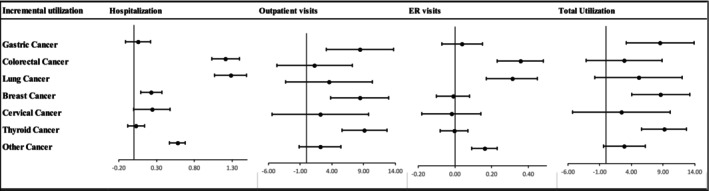
Association between cancer and incremental annual healthcare utilization stratified by cancer type. Models are adjusted for sex, age, marital status, insurance type, private health insurance (PHI) ownership, household income level, occupation, smoking status, high‐risk alcohol consumption, hypertension, and diabetes.

Figure 2 presents the results of incremental annual healthcare utilization stratified by cancer type. The analysis reveals significant differences in healthcare utilization across various cancer types after controlling for relevant factors.

For gastric cancer patients, outpatient visits increased significantly by 8.43 visits (*p* = 0.0018) and total utilization by 8.52 visits (*p* = 0.0018). Colorectal cancer patients exhibited significant differences in hospitalization (1.21 admissions; *p* < 0.0001) and ER visits (0.36 visits; *p* < 0.0001). Lung cancer patients had significant increases in hospitalization (1.28 admissions; *p* < 0.0001) and ER visits (0.31 visits; *p* < 0.0001). Breast cancer patients had significant increases in hospitalization (0.23 admissions; *p* = 0.0014), outpatient visits (8.40 visits; *p* = 0.0003), and total utilization (8.62 visits; *p* = 0.0002). Cervical cancer patients showed a significant increase in hospitalization (0.24 admissions; *p* = 0.047). Thyroid cancer patients experienced significant increases in outpatient visits (9.12 visits; *p* < 0.0001) and total utilization (9.14 visits; *p* < 0.0001). Finally, patients with other cancers had significant differences in hospitalization (0.58 admissions; *p* < 0.0001) and ER visits (0.16 visits; *p* < 0.0001).

**FIGURE 3:**
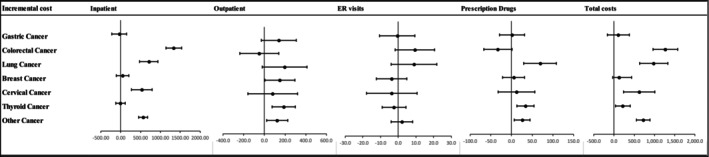
Association between cancer and incremental annual OOP costs stratified by cancer type. Models are adjusted for sex, age, marital status, insurance type, private health insurance (PHI) ownership, household income level, occupation, smoking status, high‐risk alcohol consumption, hypertension, and diabetes.

Figure 3 illustrates the annual incremental OOP expenditures by cancer type. The analysis demonstrates significant differences in OOP expenditures across various cancer types after adjusting for control variables.

Colorectal cancer patients incurred significant inpatient expenditures ($1337.1; *p* < 0.0001) and total expenditures ($1263.8; *p* < 0.0001). Lung cancer patients showed significant differences in inpatient expenditures ($706.9; *p* < 0.0001), annual medication expenditures ($69.0; *p* = 0.0006), and total expenditures ($980.0; *p* < 0.0001). Breast cancer patients had significant differences in outpatient expenditures ($149.8; *p* = 0.0429). Cervical cancer patients experienced significant differences in inpatient expenditures ($533.0; *p* < 0.0001) and total expenditures ($621.9; *p* = 0.0017). Thyroid cancer patients exhibited significant differences in outpatient expenditures ($188.4; *p* = 0.001), annual medication expenditures ($34.2; *p* = 0.0011), and total expenditures ($215.0; *p* = 0.0198).

## 4 Discussion

This study aimed to estimate the incremental annual healthcare utilization and OOP expenditures associated with cancer in South Korea. Our findings provide updated estimates, adjusted for covariates, and reveal that cancer patients experience significantly higher healthcare utilization and OOP expenditures compared to non‐cancer patients, consistent with prior research [12, 14, 25]. However, direct comparisons with other studies are challenging due to differences in health insurance policies, methodologies, and data sources.

Cancer patients had significantly more outpatient visits (4.91), hospitalizations (0.39), and emergency room (ER) visits (0.11) annually compared to non‐cancer patients. The high frequency of outpatient visits is likely driven by the need for anticancer drug treatments, radiation therapy, and diagnostic/screening services [26]. Moreover, cancer patients often experience frequent hospitalizations, particularly in the final 30 days of life [27, 28], which are typically associated with cancer‐related complications such as pain, infections, and respiratory issues [29, 30]. Similarly, the increased ER visits may be linked to acute symptoms like pain, gastrointestinal and respiratory complaints, neurological issues, fever, and bleeding [31, 32].

The incremental OOP expenditures for cancer patients was $482.8 annually, after adjusting for comorbidities and other factors. This figure contrasts with findings from the United States, where cancer patients typically face OOP expenditures ranging from $4000 to $5000 annually [14, 33]. The discrepancy can largely be attributed to South Korea's Universal Health Coverage (UHC) system, where cancer patients pay only 5%–10% of medical expenses due to the benefit extension system, compared to the higher OOP expenditures in the United States [34, 35].

Inpatient expenditures, accounting for $340.2 of the incremental OOP expenses, represent a significant portion of cancer‐related expenses, a trend observed in other countries like Canada and those in the European Union [36, 37, 38, 39, 40]. Some studies, however, suggest that outpatient expenditures are becoming the most expensive component of cancer care due to the rising expenditures of advanced therapies like immunotherapy [41, 42]. The increasing use of targeted therapies has been identified as a key driver of cancer‐related expenditures [3]. Additionally, the last year of life is often the most expensive period for cancer patients, further increasing financial burdens [43].

This study was based on 2019 data, prior to the introduction of advanced treatments like heavy ion particle cancer therapy in Korea, which could further affect future OOP expenditures. As more advanced therapies and technologies become available, it will be essential to revisit and re‐estimate the incremental expenditures associated with cancer. Furthermore, variations in patient‐specific characteristics and hospital treatment protocols suggest that a more comprehensive understanding of the direct expenditures of cancer treatment is necessary to inform policy and reduce the economic burden on patients.

While this study provides valuable insights into the healthcare utilization and OOP expenditures of cancer in Korea, several limitations must be considered. First, the data are self‐reported, which may introduce recall bias. However, the likelihood of systematic bias between cancer and non‐cancer patients is minimal. Second, the use of cross‐sectional data limits our ability to infer causal relationships between cancer, healthcare utilization, and OOP expenditures. Third, the data did not allow for detailed analysis of specific cancers like prostate cancer, and other critical factors such as cancer stage, recurrence, or time since diagnosis were unavailable. Fourth, the study did not account for indirect expenditures like productivity losses, caregiver time, or the psychological impact of cancer and direct non‐medical expenditures, such as transportation and salary stoppages to the actual healthcare expenditures. Future research should incorporate these expenditures to provide a more comprehensive assessment of the financial challenges faced by cancer patients. Lastly, this study is limited by the lack of detailed information on the treatment status of cancer patients, including whether they were undergoing active treatment, recently diagnosed, or in the post‐treatment phase. Consequently, the analysis could not account for variations in healthcare utilization or OOP expenditures across different stages of the cancer care continuum. Future studies should incorporate such data to provide a more nuanced understanding of the financial burden faced by cancer patients.

To our knowledge, this is the first study to estimate the incremental healthcare utilization and OOP expenditures associated with cancer in South Korea. The findings underscore the substantial financial burden faced by cancer patients, particularly for inpatient and outpatient services. These results highlight the need for policymakers to implement targeted strategies to alleviate the financial burdens of cancer care in Korea. Specifically, expanding the coverage of the benefit extension system to include more non‐covered services, such as advanced treatments and diagnostic procedures, could significantly reduce OOP expenditures for patients. Additionally, strengthening financial support programs for low‐income households would help address disparities in access to cancer care. Our study contributes to the growing body of research on cancer‐related healthcare expenditures and utilization and sets the stage for future studies that can offer more detailed evidence.

## 5 Conclusion

This study provides crucial estimates of the incremental economic burden associated with cancer. The findings reveal that cancer patients use all types of healthcare services more frequently than those without cancer, resulting in significantly higher expenditures. As the number of cancer survivors continues to grow, it is imperative to address the rising financial burden on this population. Clinical and public health policies should be designed to mitigate these expenditures, ensuring that cancer patients receive high‐quality care in a cost‐effective manner.

Future research should explore the broader financial strain on households with cancer patients, including catastrophic healthcare expenditures and the risk of impoverishment. Additionally, there is a need to assess the value of services and medications provided to cancer patients, aiming to optimize healthcare delivery and minimize unnecessary expenses. By focusing on cost‐effective strategies, healthcare systems can promote more efficient utilization of resources, potentially leading to long‐term expenditure savings and improved patient outcomes.

## Author Contributions

Conceptualization: Do Hee Kim and Tae Hyun Kim. Methodology: Do Hee Kim. Software: Yejin Kim and Jun Su Park. Data curation: Do Hee Kim, Yejin Kim, and Jun Su Park. Investigation: Do Hee Kim. Validation: Do Hee Kim and Tae Hyun Kim. Formal analysis: Do Hee Kim, Yejin Kim, and Jun Su Park. Supervision: Sang Gyu Lee, Hyuk‐Jae Chang, and Tae Hyun Kim. Funding acquisition: Hyuk‐Jae Chang and Tae Hyun Kim. Visualization: Do Hee Kim and Tae Hyun Kim. Project administration: Sang Gyu Lee, Hyuk‐Jae Chang, and Tae Hyun Kim. Resources: Yejin Kim and Jun Su Park. Writing – original draft: Do Hee Kim. Writing – review and editing: Sang Gyu Lee and Tae Hyun Kim.

## Conflicts of Interest

The authors declare no conflicts of interest.

## Data Availability

The KHP (https://www.khp.re.kr:444/eng/main.do) provided the raw data we used, making it publicly accessible with proper authorization. To access the raw data, you can contact the Korea Institute for Health and Social Affairs (KIHASA) or the National Health Insurance Services (NHIS).
